# Supplementary material for: Effectiveness of gamma-oryzanol in glycaemic control and managing oxidative stress, inflammation, and dyslipidaemia in diabetes: a systematic review of preclinical studies
Source: PeerJ. 2025 Sep 23;13:e20062. doi: 10.7717/peerj.20062 (PMC12466496; doi:10.7717/peerj.20062)
Supplement: Supplemental Information 1 [file peerj-13-20062-s001.docx]

**Table S1.** Search strategy

| **S/n** | **Database** | **Algorithm** | **Number of Articles retrieved** |
| --- | --- | --- | --- |
| 1 | PubMed | (Gamma-oryzanol OR γ-oryzanol OR Oryzanol OR Cycloartenyl ferulate OR Gammariza) AND (Diabetes mellitus OR Type 2 diabetes mellitus OR hyperglycaemia OR oxidative stress OR inflammation OR dyslipidaemia) | 139 |
| 2 | Scopus | *All field* (Gamma-oryzanol OR γ-oryzanol OR Oryzanol OR Cycloartenyl ferulate OR Gammariza) AND (Diabetes mellitus OR Type 2 diabetes mellitus OR hyperglycaemia OR oxidative stress OR inflammation OR dyslipidaemia) | 155 |
| 3 | Web of Science | (Gamma-oryzanol OR γ-oryzanol OR Oryzanol OR Cycloartenyl ferulate OR Gammariza) AND (Diabetes mellitus OR Type 2 diabetes mellitus OR hyperglycaemia OR oxidative stress OR inflammation OR dyslipidaemia) | 256 |
| 4 | Science Direct | i. (Gamma-oryzanol OR γ-oryzanol OR Oryzanol OR Cycloartenyl ferulate OR Gammariza) AND (Diabetes mellitus OR Type 2 diabetes mellitus OR hyperglycaemia)  ii. (Gamma-oryzanol OR γ-oryzanol OR Oryzanol OR Cycloartenyl ferulate OR Gammariza ) AND (Oxidative stress OR Inflammation OR dyslipidaemia) | 300  1139 |
| **Total** | | | **1989** |
