# Supplementary material for: Effectiveness of gamma-oryzanol in glycaemic control and managing oxidative stress, inflammation, and dyslipidaemia in diabetes: a systematic review of preclinical studies
Source: PeerJ. 2025 Sep 23;13:e20062. doi: 10.7717/peerj.20062 (PMC12466496; doi:10.7717/peerj.20062)
Supplement: Supplemental Information 2 [file peerj-13-20062-s002.docx]

|  |  |  |  |  |  |  |  | Low | Low | Low | High |
| --- | --- | --- | --- | --- | --- | --- | --- | --- | --- | --- | --- |

**Table S2.** Risk of bias assessment

1. (Chen & Cheng, 2006)

| Biases | Authors’ judgment | Support for judgment |
| --- | --- | --- |
| Sequence generation (selection bias) | High | Random generation was neither mentioned nor described in the animals’ group allocation. |
| Baseline characteristics (selection bias) | Low | The animals were of the same age, sex, and weight across all groups. The authors confirmed diabetes 14 days post-STZ injection. |
| Allocation concealment (selection bias) | Unclear | There is no clear description of whether the allocation was concealed or not. |
| Random housing (performance bias) | Unclear | The authors didn’t specify whether the animals were housed randomly. |
| Blinding of carer/administrator (performance bias) | Unclear | The authors didn’t state whether the carer was blinded or not. |
| Random outcome assessment (detection bias); All outcomes | Unclear | The authors didn’t clarify whether outcome assessment was random |
| Blinding of outcome assessor (detection bias); All outcomes | Unclear | No reported statement on the blinding of the outcome assessor |
| Incomplete outcome data adequately addressed (attrition bias); All outcomes. | Low | All animals were included in the analysis |
| Free of selective outcome reporting (reporting bias); | Low | The review team noticed no preference for data reporting. |
| Other (Other sources of bias) | High | The study is associated with units of analysis errors because γ-oryzanol was administered in combination with a diet. |

2. (Chou et al., 2009)

| Biases | Authors’ judgment | Support for judgment |
| --- | --- | --- |
| Sequence generation (selection bias) | High | Random generation was neither mentioned nor described in the animals’ group allocation. |
| Baseline characteristics (selection bias) | Low | The animals were of the same age, sex, and weight across all groups. The authors confirmed diabetes 14 days post-STZ injection. |
| Allocation concealment (selection bias) | Unclear | There is no clear description of whether the allocation was concealed or not. |
| Random housing (performance bias) | Unclear | The authors didn’t specify whether the animals were housed randomly. |
| Blinding of carer/administrator (performance bias) | Unclear | The authors didn’t state whether the carer was blinded or not. |
| Random outcome assessment (detection bias); All outcomes | Unclear | The authors didn’t clarify whether outcome assessment was random |
| Blinding of outcome assessor (detection bias); All outcomes | Unclear | No reported statement on the blinding of the outcome assessor |
| Incomplete outcome data adequately addressed (attrition bias); All outcomes. | Low | All animals were included in the analysis |
| Free of selective outcome reporting (reporting bias); | Low | The review team noticed no preference for data reporting. |
| Other (Other sources of bias) | High | The study is associated with units of analysis errors because γ-oryzanol was administered in combination with a diet. |

3. (Cheng et al., 2010)

| Biases | Authors’ judgment | Support for judgment |
| --- | --- | --- |
| Sequence generation (selection bias) | High | Random generation was neither mentioned nor described in the animals’ group allocation. |
| Baseline characteristics (selection bias) | Low | The animals were of the same age, sex, and weight across all groups.  Authors confirmed diabetes 14 days post-STZ injection. |
| Allocation concealment (selection bias) | Unclear | There is no clear description of whether the allocation was concealed or not. |
| Random housing (performance bias) | Unclear | The authors didn’t specify whether the animals were housed randomly. |
| Blinding of carer/administrator (performance bias) | Unclear | The authors didn’t state whether the carer was blinded or not. |
| Random outcome assessment (detection bias); All outcomes | Unclear | The authors didn’t clarify whether outcome assessment was random |
| Blinding of outcome assessor (detection bias); All outcomes | Unclear | No reported statement on the blinding of the outcome assessor |
| Incomplete outcome data adequately addressed (attrition bias); All outcomes. | Low | All animals were included in the analysis |
| Free of selective outcome reporting (reporting bias); | Low | No preference on data reporting was noticed. |
| Other (Other sources of bias) | High | The study is associated with units of analysis errors because γ-oryzanol was administered in combination with a diet. |

4. (Ghatak & Panchal, 2012a)

| Biases | Authors’ judgement | Support for judgement |
| --- | --- | --- |
| Sequence generation (selection bias) | High | Mentioned randomisation in grouping the animals, but did not explain the randomisation method adopted. |
| Baseline characteristics (selection bias) | High | The age of the animals is missing.  Authors confirmed DM 48 hours post-STZ injection. |
| Allocation concealment (selection bias) | Unclear | There is no clear description of whether the allocation was concealed or not. |
| Random housing (performance bias) | Unclear | The authors didn’t specify whether the animals were housed randomly. |
| Blinding of carer/administrator (performance bias) | Unclear | The authors didn’t state whether the carer was blinded or not. |
| Random outcome assessment (detection bias); All outcomes | Unclear | The authors didn’t clarify whether outcome assessment was random |
| Blinding of outcome assessor (detection bias); All outcomes | Unclear | No reported statement on the blinding of the outcome assessor |
| Incomplete outcome data adequately addressed (attrition bias); All outcomes. | Low | All animals were included in the analysis |
| Free of selective outcome reporting (reporting bias); | Low | No preference on data reporting was noticed. |
| Other (other sources of bias) | Low | Gamma oryzanol was administered in milligrams and not in combination with other bioactive compounds. |

5. (Ghatak & Panchal, 2012b)

| Biases | Authors’ judgment | Support for judgment |
| --- | --- | --- |
| Sequence generation (selection bias) | High | Mentioned randomisation in grouping the study population, but did not explain the randomisation method adopted. |
| Baseline characteristics (selection bias) | High | The age of the animals is missing.  Authors confirmed DM 72 hours post-STZ injection. |
| Allocation concealment (selection bias) | Unclear | There is no clear description of whether the allocation was concealed or not. |
| Random housing (performance bias) | Unclear | The authors didn’t specify whether the animals were housed randomly. |
| Blinding of carer/administrator (performance bias) | Unclear | The authors didn’t state whether the carer was blinded or not. |
| Random outcome assessment (detection bias); All outcomes | Unclear | The authors didn’t clarify whether outcome assessment was random |
| Blinding of outcome assessor (detection bias); All outcomes | Unclear | No reported statement on the blinding of the outcome assessor |
| Incomplete outcome data adequately addressed (attrition bias); All outcomes. | Low | All animals were included in the analysis |
| Free of selective outcome reporting (reporting bias); | Low | No preference on data reporting was identified. |
| Other (other sources of bias) | Low | Gamma oryzanol was administered in milligrams and not in combination with other bioactive compounds. |

6. (Ghatak & Panchal, 2014)

| Biases | Authors’ judgment | Support for judgment |
| --- | --- | --- |
| Sequence generation (selection bias) | High | Mentioned randomisation in grouping the study population, but did not explain the randomisation method adopted. |
| Baseline characteristics (selection bias) | High | The age of the animals is missing. Diabetes was confirmed 48 hours post-STZ injection. |
| Allocation concealment (selection bias) | Unclear | There is no clear description of whether the allocation was concealed or not. |
| Random housing (performance bias) | Unclear | The authors didn’t specify whether the animals were housed randomly. |
| Blinding of carer/administrator (performance bias) | Unclear | The authors didn’t state whether the carer was blinded or not. |
| Random outcome assessment (detection bias); All outcomes | Unclear | The authors didn’t clarify whether outcome assessment was random |
| Blinding of outcome assessor (detection bias); All outcomes | Unclear | No reported statement on the blinding of the outcome assessor |
| Incomplete outcome data adequately addressed (attrition bias); All outcomes. | Low | All animals were included in the analysis |
| Free of selective outcome reporting (reporting bias); | Low | No preference on data reporting was identified. |
| Other (other sources of bias) | Low | Gamma oryzanol was administered in milligrams and not in combination with other bioactive compounds. |

7. (Kozuka et al., 2017)

| Biases | Authors’ judgement | Support for judgement |
| --- | --- | --- |
| Sequence generation (selection bias) | High | Random generation was neither mentioned nor described in the animals’ group allocation. |
| Baseline characteristics (selection bias) | Low | The animals were of the same age, sex, and weight across all groups.  Genetically *ob/ob* mice can exhibit signs of diabetes as early as 5 weeks of age, with severe diabetes occurring around 8 weeks of age. In this experiment, diabetes was confirmed at 5 weeks, and treatment was executed for 4 weeks, making a total of 9 weeks. |
| Allocation concealment (selection bias) | Unclear | There is no clear description of whether the allocation was concealed or not. |
| Random housing (performance bias) | Unclear | The authors didn’t specify whether the animals were housed randomly. |
| Blinding of carer/administrator (performance bias) | Unclear | The authors didn’t state whether the carer was blinded or not. |
| Random outcome assessment (detection bias); All outcomes | Unclear | The authors didn’t clarify whether outcome assessment was random |
| Blinding of outcome assessor (detection bias); All outcomes | Unclear | No reported statement on the blinding of the outcome assessor |
| Incomplete outcome data adequately addressed (attrition bias); All outcomes. | Low | All animals were included in the analysis |
| Free of selective outcome reporting (reporting bias); | Low | No preference on data reporting was identified. |
| Other (other sources of bias) | Low | Gamma oryzanol was administered in milligrams and not in combination with other bioactive compounds. |

8. (G et al., 2018)

| Biases | Authors’ judgement | Support for judgement |
| --- | --- | --- |
| Sequence generation (selection bias) | High | Random generation was neither mentioned nor described in the animals’ group allocation. |
| Baseline characteristics (selection bias) | High | The authors didn’t report the animals’ age, but interestingly, diabetes was confirmed 7 days after STZ injection |
| Adequate timing of disease induction (selection bias) | Low | Diabetes was confirmed 7 days after STZ injection |
| Allocation concealment (selection bias) | Unclear | There is no clear description of whether the allocation was concealed or not. |
| Random housing (performance bias) | Unclear | The authors didn’t specify whether the animals were housed randomly. |
| Blinding of carer/administrator (performance bias) | Unclear | The authors didn’t state whether the carer was blinded or not. |
| Random outcome assessment (detection bias); All outcomes | Unclear | The authors didn’t clarify whether outcome assessment was random |
| Blinding of outcome assessor (detection bias); All outcomes | Unclear | No statement was reported on the blinding of the outcome assessor |
| Incomplete outcome data adequately addressed (attrition bias); All outcomes. | Low | All animals were included in the analysis |
| Free of selective outcome reporting (reporting bias); | Low | No preference on data reporting was identified. |
| Other (other sources of bias) | High | Gamma oryzanol was administered with a diet, which is associated with unit of analysis errors. |

9. (Bhaskaragoud et al., 2020)

| Biases | Authors’ judgement | Support for judgement |
| --- | --- | --- |
| Sequence generation (selection bias) | High | Random generation was neither mentioned nor described in the animals’ group allocation. |
| Baseline characteristics (selection bias) | High | The authors didn’t report the animals’ age and time of diabetes confirmation post-STZ injection. |
| Allocation concealment (selection bias) | Unclear | There is no clear description of whether the allocation was concealed or not. |
| Random housing (performance bias) | Unclear | The authors didn’t specify whether the animals were housed randomly. |
| Blinding of carer/administrator (performance bias) | Unclear | The authors didn’t state whether the carer was blinded or not. |
| Random outcome assessment (detection bias); All outcomes | Unclear | The authors didn’t clarify whether outcome assessment was random |
| Blinding of outcome assessor (detection bias); All outcomes | Unclear | No reported statement on the blinding of the outcome assessor |
| Incomplete outcome data adequately addressed (attrition bias); All outcomes. | High | It is unclear whether all animals were included in the analysis. While grouping the animals, 6 and 10 were allocated for negative and positive controls, but only six were included in the analysis of some parameters and not mentioned for others. |
| Free of selective outcome reporting (reporting bias); | Low | No preference on data reporting was identified. |
| Other (other sources of bias) | High | Gamma oryzanol was administered with a diet, which is associated with unit of analysis errors. |
