# Supplementary material for: Effectiveness of gamma-oryzanol in glycaemic control and managing oxidative stress, inflammation, and dyslipidaemia in diabetes: a systematic review of preclinical studies
Source: PeerJ. 2025 Sep 23;13:e20062. doi: 10.7717/peerj.20062 (PMC12466496; doi:10.7717/peerj.20062)
Supplement: Supplemental Information 3 [file peerj-13-20062-s003.docx]

**Table S3.** Excluded full-text articles with reasons (n = **4**)

| **Studies** | **Reasons** |
| --- | --- |
| (Kozuka et al., 2015) | No related outcome measures |
| (Adamu et al., 2017), (Francisqueti et al., 2018), and (Mattei et al., 2021) | Inappropriate disease model |
